# Assessing the Biogeographic Risks of Potentially Toxic Plants—A Case Study for a Novel Locoweed *Sphaerophysa salsula* in China

**DOI:** 10.1002/ece3.73074

**Published:** 2026-02-11

**Authors:** Yue‐Yang Zhang, Hua‐Qi Liu, Tong‐Tong Wang, Ya‐Na Wang, Yan‐Zhong Li

**Affiliations:** ^1^ State Key Laboratory of Herbage Improvement and Grassland Agro‐Ecosystems, College of Pastoral Agriculture Science and Technology, Center for Grassland Microbiome Lanzhou University; Engineering Research Center of Grassland Industry, Ministry of Education Lanzhou China

**Keywords:** climate change, endophyte, livestock poisoning, locoweed, MaxEnt, poisonous plants

## Abstract

Climate change‐induced grassland degradation has exacerbated the spread of toxic plants, yet many potentially toxic species remain overlooked, undermining rangeland management and causing significant economic losses. Quantifying the toxicity and distribution of potential toxic plants under climate change is critical for mitigating biogeographic risks. As a case study, taking *Sphaerophysa salsula* , a leguminous plant distributed in Asia and the Americas, historically utilized for erosion control but recently associated with livestock poisoning, this research integrated toxicity identification, species distribution modeling (SDM), and risk assessment to evaluate its biogeographic threats in China. Results suggested for the first time that *S. salsula* can function as a high‐toxicity (chemotype 1) locoweed due to swainsonine (mean content 0.373%), produced by its endophyte *Alternaria oxytropis* (23.46 pg/ng), which is implicated in locoism‐like syndromes in livestock. The Maximum Entropy model identified temperature annual range (43.22°C), mean temperature of the driest quarter (−6.29°C), and soil pH (8.61) as key distribution drivers. Currently, suitable habitats are concentrated in Northern China (Xinjiang, Inner Mongolia, Ningxia). By the 2070s, these habitats are projected to decline by 6.3%–9%, shifting westward toward pastoral regions. Risk assessments integrating grazing intensity revealed high‐risk zones in Gansu, Ningxia, and Inner Mongolia, with future scenarios predicting declining risks in eastern Inner Mongolia but increasing threats in western Tibet. These findings clarify *S. salsula* 's toxic mechanism and biogeographic risks, providing a framework for targeted management of overlooked toxic plants under climate change.

## Graphical abstract

This study firstly identified the toxic principles of *S. salsula* and proposed a MaxEnt model to predict its suitable habitats and assess livestock poisoning risks under various SSP scenarios.

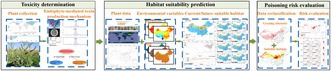

## 1 Introduction

The expansion of plants that are toxic to grazing animals has become a global concern (Holechek 2002; Michael 2002; Zhang et al. 2017). Climate change and extreme events (precipitation, droughts, floods, storms, and permafrost thaw/loss) accelerate grassland degradation, creating favorable conditions for toxic weeds while suppressing dominant forage species (VanDerWal et al. 2013; Yuan et al. 2021; Zhang, Sun, et al. 2025). For example, in China alone, approximately 1300 toxic plant species across 140 families infest 33.3 million hectares of natural grasslands, causing annual economic losses exceeding billions of CNY (Zhang et al. 2020). This crisis directly undermines the United Nations Sustainable Development Goals, particularly “Zero Hunger” and “No Poverty” (Blesh et al. 2019).

Despite their significant harm, many toxic plants in grasslands remain poorly understood due to ecosystem complexity, with some even being misused for their ecological adaptability (Bala et al. 2024; Zhang, Seabloom, et al. 2025; Zhang et al. 2020). *Sphaerophysa salsula* , a perennial legume native to Asia, is a striking example (Qu et al. 2021). It is adapted to arid environments and saline‐alkaline soils. Studies have shown that planting *S. salsula* can improve saline‐alkaline soils by increasing organic matter and nutrient content (Wang et al. 2021). Its root nodules also host diverse bacterial communities, including nitrogen‐fixing rhizobia, which facilitate these soil interactions (Deng et al. 2011). Given its combined ecological traits, *S. salsula* was originally introduced to the western United States for erosion control (Ditomaso and Kyser 2013). However, it has since become invasive and is now officially designated as a noxious weed in multiple states (Ditomaso and Kyser 2013). In its native range, however, the plant's utility took a different form: in China, it has been traditionally used as winter forage during herbage shortages despite emerging evidence of its toxicity (Deng et al. 2011; Region 1990). Most troubling are reports linking *S. salsula* to livestock poisoning incidents in China, with clinical symptoms remarkably similar to locoism (Yan et al. 2014). Locoism, a chronic condition caused by *Astragalus* and *Oxytropis* spp. (locoweed), induces neurological disturbances, emaciation, addictive effects, and reproductive disturbances in livestock (Colegate et al. 1979; Fu et al. 2023; Hartley 1978). This toxicity stems from the indolizidine alkaloid swainsonine, synthesized by endophyte *Alternaria* sect. *Undifilum* (Cook et al. 2021; Fu et al. 2024; Grum et al. 2013; James et al. 1967). Mass sheep mortality events in Qinghai and Xinjiang by *S. salsula* highlight its threat, yet its toxic mechanism remains unresolved (Yan et al. 2014). We hypothesize that like locoweeds, *S. salsula* may harbor swainsonine‐producing endophytes, necessitating urgent toxicity determination and biogeographic risk assessment.

Accurately predicting the distribution patterns of toxic plants under evolving climatic conditions is essential for developing effective strategies to protect livestock health and maintain grassland ecosystem integrity (Cook et al. 2021). Despite studies on *S. salsula* 's phytochemistry (Lee et al. 2021), its distribution dynamics and climate‐driven range shifts remain unstudied, impeding risk management. Species distribution models (SDMs) are vital approaches that can predict species distributions under changing climates (Parmesan and Yohe 2003). While several SDMs like Genetic Algorithm for Rule‐Set Prediction model (Buebos‐Esteve et al. 2023) and CLIMate EXpert distribution model (Yoon and Lee 2023) exist, the Maximum Entropy (MaxEnt) model outperforms others in accuracy and flexibility, particularly for rare or toxic species (Fois et al. 2018; Phillips et al. 2006). MaxEnt's leverage of limited occurrence data and diverse environmental variables allows for the generation of robust predictions and makes it ideal for this study (Kaky et al. 2020; Wisz et al. 2008; Zhao et al. 2024).

Here, four key questions were raised: (1) Does *S. salsula* contain swainsonine or swainsonine‐producing endophytes? (2) What is the current distribution of suitable habitats for *S. salsula* ? (3) How will the suitable habitats of *S. salsula* shift under future climate scenarios? (4) How will the risk of livestock poisoning by *S. salsula* change under climate change? By combining toxicity studies (Qinghai populations), MaxEnt‐based habitat modeling, and livestock poisoning risk assessment, we aim to quantify biogeographic risks of the potential toxic plant *S. salsula* and inform control strategies.

## 2 Materials and Methods

### 2.1 Plant Material

**FIGURE 1:**
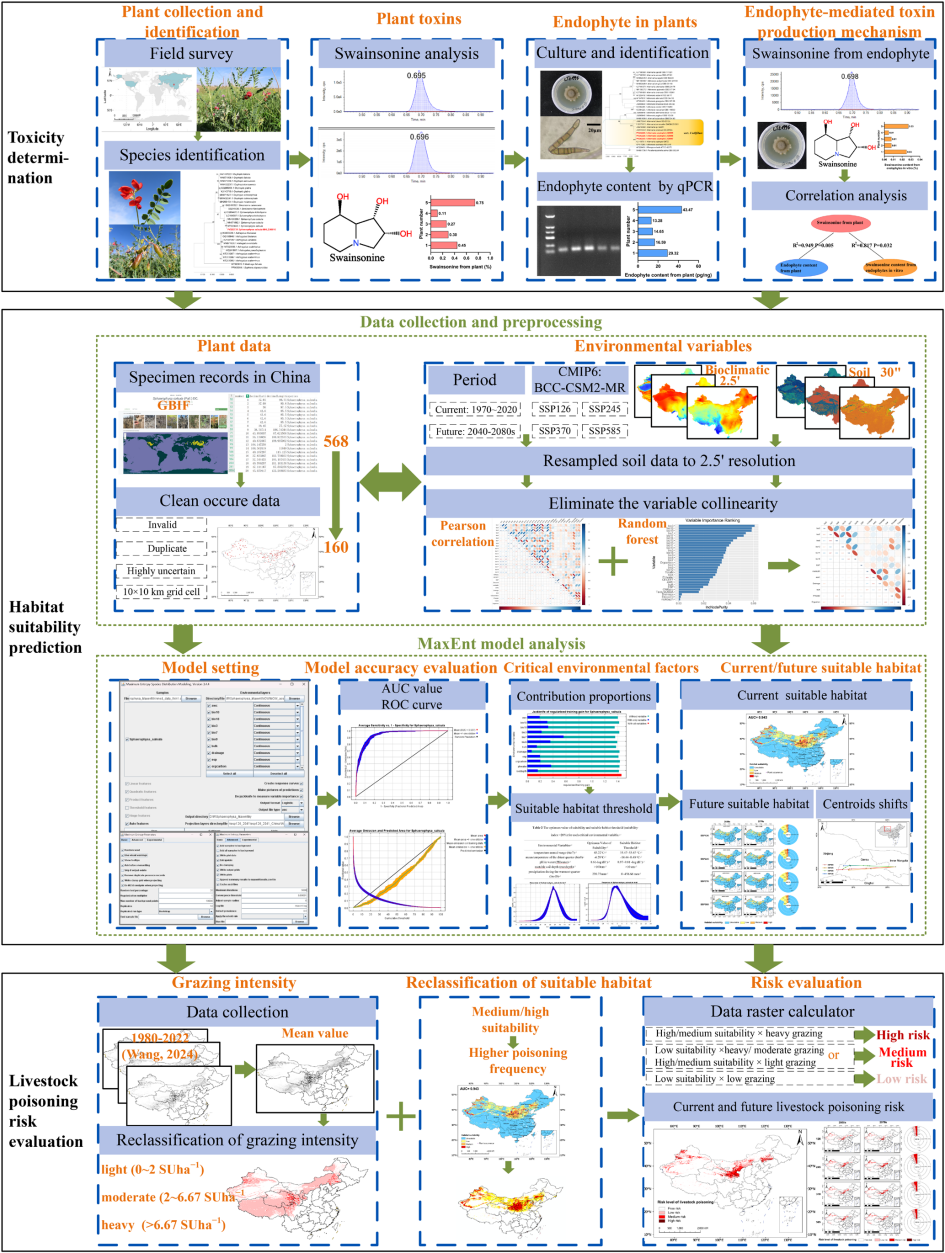
The framework of this study's contribution.

*Sphaerophysa salsula* samples were collected from Haixi Mongolian and Tibetan autonomous prefecture, Qinghai province, China (N 37.00°, E 98.20°) during July 2023 (Figure 1). A total of 15 individual plants were collected at the site. The plant specimens were stored in the specimen library of Lanzhou University, numbered MHLZU6816. From these, five representative plants were selected based on visual health and spatial distribution across the sampling area for detailed analysis, and 2 cm of stem segments were cut for endophyte culture. Then, the remainder were freeze‐dried and ground to extract swainsonine and DNA for further analyses.

### 2.2 Plant Taxonomic Identification

Morphological identification was conducted by Prof. Yuhu Wu from the Northwest Institute of Plateau Biology, Chinese Academy of Sciences, an expert in Fabaceae taxonomy.

For molecular identification, DNA of *S. salsula* was extracted from the tissue using the Ezup Column Plant Genomic DNA Extraction Kit (Sangon Biotech, Shanghai, China). DNA concentrations were measured using a NanoDrop ND‐1000 Spectrophotometer (NanoDrop Technologies, Wilmington, Delaware, USA), and aliquots were stored at −20°C until use.

PCR amplification was performed using ITS1‐f/ITS4‐r primers (Baldwin and Markos 1998). The PCR products were detected by agarose gel electrophoresis (120 V, 400 mA, 30 min) using a Bio‐Rad PowerPac Basic electrophoresis instrument (Bio‐Rad Laboratories, California, USA). DNA bands (700 bp) were visualized with a gel imaging machine (GelStudio, Analytik Jena US LLC, California, USA). PCR products were sent to Sangon Biotech for sequencing. Sequence data were processed using software SeqMan v7.1.0 (DNASTAR, Madison, WI, USA), and spliced sequences were compared with those in the National Center for Biotechnology Information (NCBI) database (https://www.ncbi.nlm.nih.gov/). A phylogenetic tree was constructed using the Maximum Likelihood method in MegaX software (Mega Limited, Auckland, New Zealand). The newly generated plant sequence was deposited in the NCBI database. Newly generated plant sequence and reference sequences used in this study were listed in Table S1.

### 2.3 Endophyte Cultures

Stem segments were washed under running water to remove the surface debris, followed by sterilization as described by Liu et al. (2020). Small tissue pieces (about 3 mm) were placed on potato dextrose agar (PDA) medium and incubated at 22°C. Endophyte growth was observed after 24 h. The criteria for determining *Alternaria* Section *Undifilum* were (1) slow‐growing mycelium originating from the incision, and (2) white, curved, and wavy mycelium morphology. If *Alternaria* Section *Undifilum* grew on any of the stem segments, the individual plant was considered to host the endophyte.

Nine fungal cultures were subcultured, with a single pinpoint mass of mycelium placed on the PDA. The colony morphology and conidia of cultures were observed using a Nikon Optiphot microscope (Nikon, Tokyo, Japan) after 40 days of incubation. After 40 days, the cultures were freeze‐dried and weighed for further swainsonine analyses.

### 2.4 Swainsonine Analysis

Swainsonine extraction was performed by acetic acid as described by Gardner and Cook (2011). Dried plant material (50 mg) or freeze‐dried mycelia were extracted in 2% acetic acid for 18 h with agitation. After extraction, samples were centrifuged and an aliquot from the extraction was diluted into 20 mM ammonium acetate in a 1 mL autosampler vial. A certified swainsonine standard was used to prepare a series of working solutions through serial dilution. A calibration curve was established over the concentration range of 0.2–200 μg/L, which showed excellent linearity with a correlation coefficient (*R* ^2^) of 0.9979. Method blanks (extraction without a sample) were processed with each batch to monitor potential contamination.

Samples were analyzed by UPLC–MS/MS to quantify swainsonine as follows. Chromatographic conditions: (a) column: CAPCELLPAK C18 (100 mm × 2.1 mm, 2 μm); (b) mobile phase: A: 5% methanol in water, B: 20 mM ammonium acetate in water; (c) gradient elution: 0–0.5 min, 90% A; 0.5–3 min, 90% A → 5% A; (d) flow rate: 0.3 mL/min; (e) column temperature: 30°C; (f) injection volume: 2 μL. Mass conditions: (a) ion source: ESI+; (b) scan mode: positive ion mode, multiple reaction monitoring (MRM); (c) ion source voltage: 5.5 kV; (d) ion source temperature: 550°C; (e) curtain gas pressure: 241.3 kPa; (f) collision gas pressure: medium; (g) nebulizer gas pressure (GS1) and auxiliary heating gas pressure (GS2): 344.7 kPa. Quantitative ion pairs for kusnezovine: m/z 174.3/156.0, collision energy 19 V; qualitative ion pairs: *m*/*z* 174.3/138.2, collision energy 26 V; optimized declustering potential (DP): 60 V.

### 2.5 Determination of Endophyte Concentration of *S. salsula* Plants

A quantitative real‐time PCR method (qPCR) was used to determine the amount of the endophytic DNA from *S. salsula* plant, which was extracted for molecular identification (Cook et al. 2009). For absolute quantification, a standard curve was constructed using a 10‐fold serial dilution (2.3–2.3 × 10^−8^ ng/μL) of genomic DNA from a pure fungal culture, with each dilution analyzed in quadruplicate. The resulting standard curve (*Y* = −3.413*X* + 27) showed excellent linearity (*R* ^2^ = 0.990). Plant DNA samples (Section 2.3), normalized to 50 ng/μL, were analyzed in triplicate using the primers SwnK‐q‐S/SwnK‐q‐AS (Guo et al. 2022) in a 10 μL SYBR Green reaction system. Each qPCR run included no‐template controls (NTCs) to monitor contamination and was followed by melt curve analysis. Runs were considered valid only when the standard curve met preset criteria for amplification efficiency (derived from the slope) and linearity (*R* ^2^ > 0.98). The endophyte DNA concentration in plant samples (pg/ng plant DNA) was calculated by interpolating the mean Cq value into the validated standard curve. Pearson correlation analysis was done using GraphPad Prism 10.0.0 (GraphPad Software, Boston, MA, USA).

### 2.6 Molecular Taxonomic Identification of Endophytes

DNA of nine endophyte strains isolated from *S. salsula* was extracted using the HP Fungal DNA Kit (Omega Bio‐Tek, Georgia, USA). PCR amplification was performed using ITS1‐f/ITS4‐r primers (White et al. 1990). To do this, an initial denaturation step for 5 min at 94°C, followed by 35 cycles of 30 s at 94°C, 45 s at 54°C, and 1 min at 72°C, with a final extension of 10 min at 72°C. The amplified products were sequenced by Sangon Biotech. Raw sequences were assembled and edited using SeqMan v7.1.0, followed by sequence homology analysis through BLASTn searches against the NCBI GenBank database with ≥ 97% similarity threshold. UPGMA phylogenetic tree was constructed with 1000 bootstrap replicates in MEGAX software. All newly generated *A. oxytropis* sequences were deposited in the NCBI database, and newly generated and referenced sequences used in this study were also listed in Table S2.

### 2.7 Species Occurrence Data and Environmental Variables Used in the MaxEnt Model

Species occurrence data for *S. salsula* in China were obtained from the Global Biodiversity Information Facility (www.gbif.org/). We collected a total of 568 data points on the occurrence of *S. salsula* in China. These distribution data were filtered using “CoordinateCleaner” and “spThin” in R v4.3 to ensure that each 10 × 10 km grid cell contained only one record (Aiello‐Lammens et al. 2015), a standard distance used to mitigate spatial sampling bias and autocorrelation in ecological niche modeling (Boria et al. 2014). This process ultimately gave 160 occurrences for subsequent analysis.

We selected 31 environmental variables for the model to predict the probability distributions of *S. salsula* , including 19 biological variables and 12 soil variables (Table 1). More specifically, to depict the current climatic situation, 19 biological variables from the WorldClim database, version 2.0 (https://www.worldclim.org/), were used with a spatial resolution of 5 min, while the Harmonized World Soil Database (https://gaez.fao.org/pages/hwsd) provided the 12 soil variables.

**TABLE 1:** Information of 31 environmental bioclimatic for the MaxEnt model in paper.

Variables class	Abbreviation	Full name
Biological variables	bio1	Annual mean temperature (°C)
	bio2	Mean diurnal temperature range (°C)
	bio3^a^	Isothermality^a^
	bio4	Temperature seasonality
	bio5	Maximum temperature of warmest month (°C)
	bio6	Minimum temperature of coldest month (°C)
	bio7^a^	Temperature annual range (°C)^a^
	bio8	Mean temperature of wettest quarter (°C)
	bio9^a^	Mean temperature of driest quarter (°C)^a^
	bio10^a^	Mean temperature of warmest quarter (°C)^a^
	bio11	Mean temperature of coldest quarter (°C)
	bio12	Annual precipitation (mm)
	bio13	Precipitation of wettest month (mm)
	bio14	Precipitation of driest month (mm)
	bio15	Precipitation seasonality (mm)
	bio16	Precipitation of wettest quarter (mm)
	bio17	Precipitation of driest quarter (mm)
	bio18^a^	Precipitation of warmest quarter (mm)^a^
	bio19	Precipitation of coldest quarter (mm)
Soil variables	bulk^a^	Bulk density (g/cm^3^)^a^
	CNRatio	C/N Ratio
	CECEFF	Effective cation exchange capacity (cmolc/kg)
	ESP^a^	Exchangeable sodium percentage^a^
	eleccond	Electric conductivity (dS/m)
	rootdepth^a^	Rootable soil depth class^a^
	AWC^a^	Available water capacity (mm)^a^
	drainage^a^	Reference soil drainage^a^
	TextureUSDA	Texture class (USDA convention)
	phwater^a^	pH in water (−log (H+))^a^
	TotalN	Total nitrogen content (g/kg)
	Orgcarbon^a^	Organic carbon content (g/kg)^a^

^a^ The variables ultimately used for modeling in paper.

Future climate scenarios were projected using four Shared Socioeconomic Pathways (SSPs) and the BCC‐CSM2‐MR model from the Beijing Climate Center, confirmed as highly effective for simulating climate variations in China (Xin et al. 2019).

Many variables exhibit spatial collinearity, which can lead a model to suffer from overfitting and ultimately impact the prediction outcomes (Veloz 2009). First, Pearson correlation analysis on all 31 environmental variables was calculated using R. Variables with an absolute correlation coefficient |*r*| ≥ 0.8 were identified as highly correlated. Then, among each group of correlated variables, we retained the one with the highest importance score derived from a preliminary random forest model and removed the others (Fang et al. 2021). This stepwise process was repeated until all remaining variables showed |*r*| < 0.8. This meant that, ultimately, five climatic variables and seven soil variables were selected to participate in the model (Table 1). Finally, we used the ArcGIS resampling tool to resample the resolution of the 12 selected environmental variable layers to 1 km for model analysis.

### 2.8 MaxEnt Model Analysis

To predict the possible habitat distributions of *S. salsula* in China under climate change, the MaxEnt model, which is a model based on the theory of maximum entropy, was selected by MaxEnt version 3.4.4 (Abdelaal et al. 2019). The training and test data points were 75% and 25%, respectively. The settings selected were as follows: “Create response curves”, “Do jackknife to measure variable importance”, “Random seed”, “Write plot data”, “Write background predictions”, “Max number of background points: 10000”, “Maximum iterations: 5000”, “Replicated run type: bootstrap”, “Replicates: 10” and “Output format logistic”. The rest of the settings were default. Model performance was evaluated using two complementary metrics: the Area Under the Receiver Operating Characteristic Curve (AUC) and the True Skill Statistic (TSS). The AUC provides a threshold‐independent measure of the model's overall discrimination ability, with values ranging from 0 to 1 (Boria et al. 2014). Typically, an AUC > 0.9 indicates excellent predictive performance, while 0.8 < AUC < 0.9 denotes good performance (Williams et al. 2009). TSS ranges from −1 to 1, with the following interpretations: poor (−1 to 0.4), fair (0.4–0.5), good (0.5–0.7), very good (0.7–0.85), excellent (0.85–0.9), and nearly perfect to perfect (0.9–1) (Allouche et al. 2006).

### 2.9 Habitat Suitability Classification and Visualization of Future Changes

The ASC files output by MaxEnt were imported into ArcGIS, converted into grid data, and superimposed on China's administrative zoning map for visual processing. In this study, we employed a manual classification method, which involves defining fixed thresholds to discretize the continuous suitability index. This approach, as opposed to data‐driven automated classifications (e.g., natural breaks), ensures consistency and facilitates cross‐study comparison (Shi et al. 2023). Specifically, the suitability index (P) was categorized into four grades: high suitability area (*p* ≥ 0.6), medium suitability area (0.4 ≤ *p* < 0.6), low suitability area (0.2 ≤ *p* < 0.4), and unsuitable area (*p* < 0.2).

### 2.10 Evaluate of the Risk of Livestock Poisoning Based on Habitat Suitability and Grazing Intensity

The risk of livestock poisoning is jointly influenced by habitat suitability and grazing intensity (Holechek 2002; Huang et al. 2022). The frequency of incidence of livestock poisoning in the medium and high suitability habitat was 66% higher than that in low suitability habitat (Huang et al. 2022), while livestock death losses to poisonous plants under heavy grazing (4.8%) were higher than those under moderate grazing intensities (2.0%) (Holechek 2002). The grazing intensity data used in this study were extracted from the 1980–2020 period of the Long‐term High‐resolution Grazing Intensity (LHGI) dataset for China (Wang, Peng, et al. 2024), which provides a spatial resolution of 0.1 × 0.1° for this interval. This temporal range was selected to maintain consistency with the baseline period of the climate variables used in our MaxEnt model. Due to the different grazing intensity classification criteria for different types of grasslands, the cross‐grassland‐type grazing intensity classification criterion summarized by Gu et al. (2023) was adopted: light grazing intensities (0–2 SU ha^−1^, excluding 0); moderate grazing intensities (2–6.67 SU ha^−1^, excluding 2); heavy grazing intensities (> 6.67 SU ha^−1^).

Based on habitat suitability and grazing intensity, the risk of livestock poisoning to *S. salsula* in current and future was classified by ArcGIS as: high risk (high/medium habitat suitability × heavy grazing intensities); medium risk (low habitat suitability × heavy/moderate grazing intensities or high/medium habitat suitability × light grazing intensities); low risk (low habitat suitability × low grazing intensities). The remaining areas are risk‐free zones.

## 3 Results

### 3.1 Swainsonine and Endophyte Determination in *Sphaerophysa salsula* Plants

**FIGURE 2:**
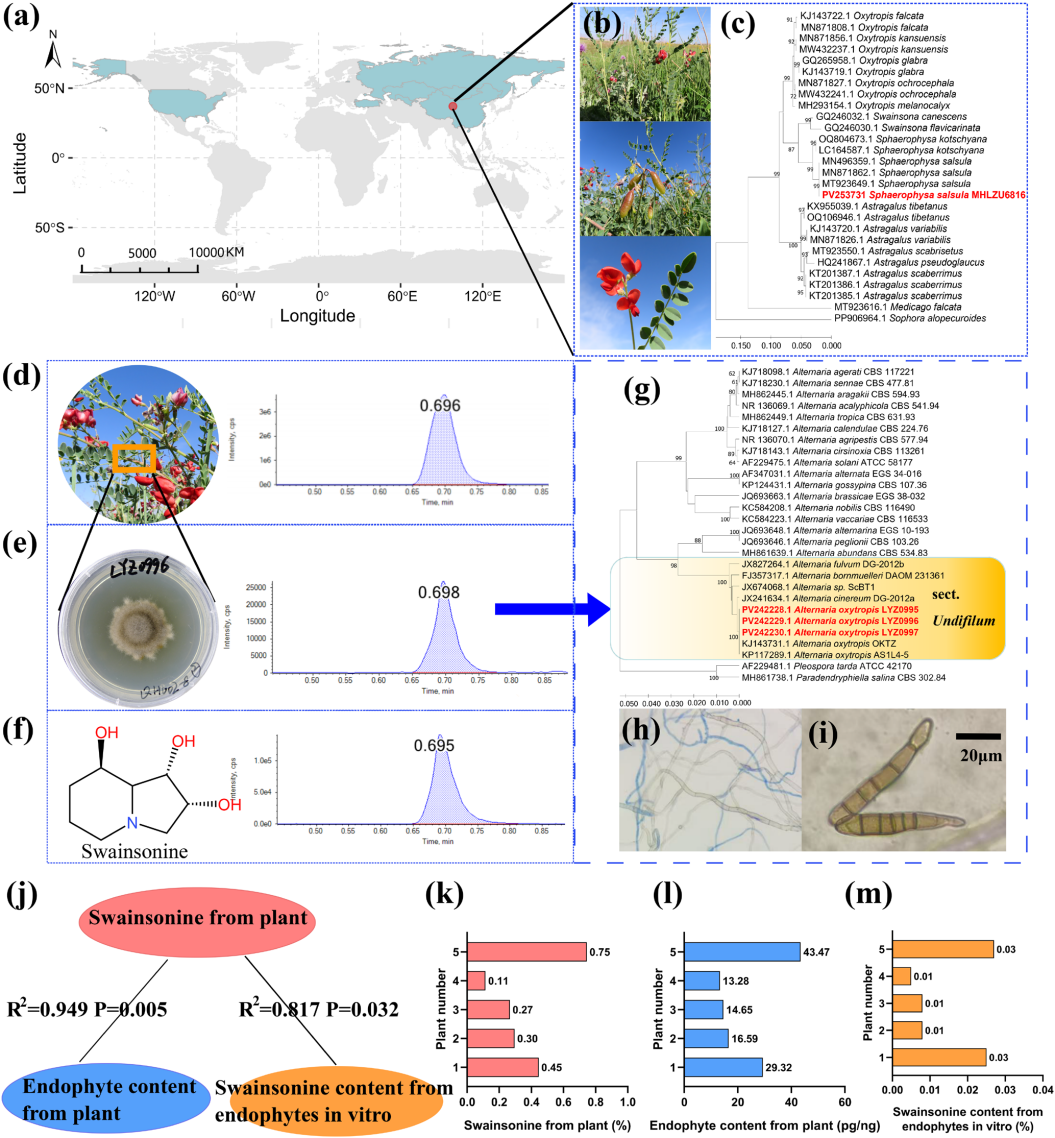
Endophyte‐mediated swainsonine toxicity in *Sphaerophysa salsula* . (a) Global distribution and sampling locations of *S. salsula* ; (b) Morphological characteristics of *S. salsula* ; (c) Molecular identification of *S. salsula* (maximum likelihood phylogenetic tree based on internal transcribed spacer [ITS] rDNA); (d) Multiple reaction monitoring (MRM) chromatogram of *S. salsula* plant extract; (e) MRM chromatogram of endophyte culture; (f) MRM chromatogram of swainsonine standard; (g) Molecular identification of the endophyte *Alternaria oxytropis* (UPGMA phylogenetic tree based on ITS rDNA); (h) Septate hyphae of *A. oxytropis* under microscopy; (i) Conidia of *A. oxytropis* (dark brown, long‐ellipsoid, 8–10 × 42–56 μm, with 4–5 transverse septa); (j) Pearson correlation analysis among: Swainsonine content in *S. salsula* plants (k), endophyte colonization density in plants (l), and swainsonine production by endophytes in vitro (m).

A patch of red‐flowered leguminous plants with oval pods was found on the plains grassland of Qinghai province (Figure 2a–c). Morphological identification confirmed that the plants belonged to *Sphaerophysa salsula* . Furthermore, phylogenetic analysis based on ITS sequences demonstrated that the species is *S. salsula* , supported by a high bootstrap value of 99, thereby validating the accuracy of the taxonomic classification (Figure 2c).

The *S. salsula* plants contained swainsonine as verified by UPLC–MS/MS (Figure 2d,f). The mean concentration of swainsonine in *S. salsula* was 0.373%, with a range of 0.114%–0.745% (Figure 2k).

Endophytes were successfully detected from all five *S. salsula* plant stems (Figure 2e). The mean concentration of endophyte from *S. salsula* was 23.46 pg/ng, with a range of 13.28–43.47 pg/ng (Figure 2m).

### 3.2 Taxonomic and Swainsonine Determination of Endophytes From *S. salsula* Plants

Endophytes from *S. salsula* grew very slowly on PDA media, with a colony diameter reaching only 6 mm after more than 30 days of incubation (Figure 2e). Colonies were composed of septal hyphae (Figure 2h). The conidia of the endophyte were long, ellipsoid in shape, straight, with 4–5 transverse septa, dark‐brown in color, and their dimensions were 8–10 × 42–56 μm (Figure 2i). The endophytes were identified as *Alternaria* Section *Undifilum* based on their morphology (Figure 2h,i). Furthermore, it was confirmed to be *Alternaria oxytropis* species by the molecular analysis of the internal transcribed spacer rDNA (ITS) (Figure 2g). The ITS sequences obtained from the nine isolates of the endophyte have been deposited with GenBank (PV242228‐PV242236).

The endophytes isolated from *S. salsula* all produced swainsonine in vitro (Figure 2e). The mean concentration of swainsonine for endophytes was 0.0146%, with a range of 0.005%–0.027% (Figure 2m). The content of swainsonine in *S. salsula* plants was significantly correlated with the endophytes' content of plants (*R* ^2^ = 0.949, *p* = 0.005) and the swainsonine content from endophyte (*R* ^2^ = 0.817, *p* = 0.032) (Figure 2j).

### 3.3 Key Environmental Variables Used in MaxEnt Model

**FIGURE 3:**
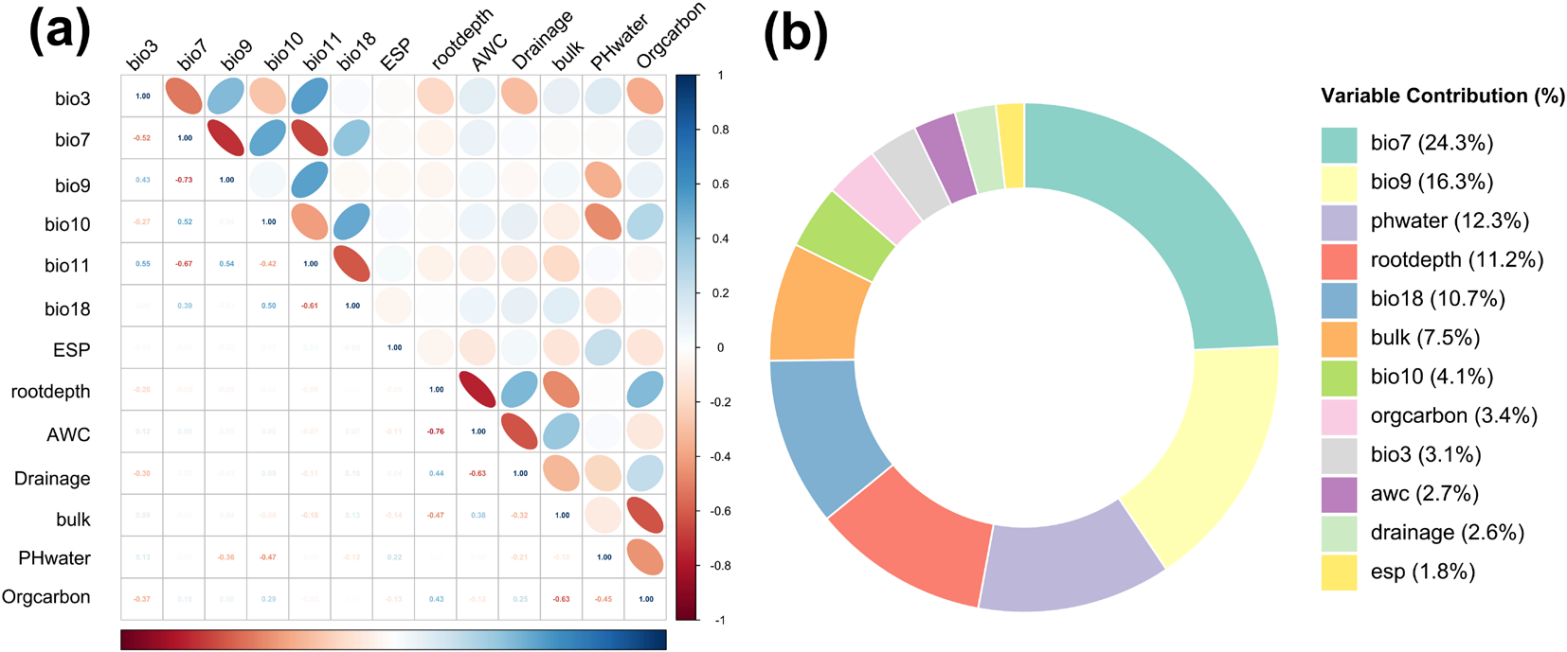
Heatmap of the variables included in the MaxEnt model (a) and these variables' contribution to the model (b).

The importance of environmental and soil factors was assessed using random forest analysis (Figure S1), from which 12 variables with |*r*| < 0.8 were selected for subsequent modeling (Figure 3a). In the MaxEnt model, five variables exhibited the highest contribution rates (Figure 3b): temperature annual range (bio7, 24.3%), mean temperature of driest quarter (bio9, 16.3%), pH in water (PHwater, 12.3%), rootable soil depth (rootdrpth, 11.2%), and precipitation of warmest quarter (bio18, 10.7%). Collectively, these five factors accounted for 74.8% of the total contribution, indicating their predominant role in governing the distribution of *S. salsula* .

Analysis of response curves (Figure S2, Table 2) demonstrated that optimal growth conditions of *S. salsula* occurred at 43.22°C, with a suitable range of 35.47°C–53.67°C for the temperature annual range (bio7). The optimal growth condition for *S. salsula* occurred when the mean temperature of the driest quarter (bio9) reached −6.29°C. Soil preferences included slightly alkaline conditions (pH in water optimum at 8.61) and deep soils (> 100 cm rootable soil depth). Suitability of *S. salsula* peaked at 239.73 mm precipitation during the warmest quarter (bio18).

**TABLE 2:** The optimum value of suitability and suitable habitat threshold (suitability index > 20%) for each critical environmental variable.

Environmental variables	Optimum value of suitability	Suitable habitat threshold
Temperature annual range (bio7)	43.22°C	35.47°C to 53.67°C
Mean temperature of the driest quarter (bio9)	−6.29°C	−14.44°C to 8.40°C
Ph in water (phwater)	8.61	0.97–9.81
Rootable soil depth (rootdepth)	> 100 cm	> 10 cm
Precipitation during the warmest quarter (bio18)	239.73 mm	0–456.66 mm

### 3.4 Potential Distribution of *S. salsula* in the Current Climate

**FIGURE 4:**
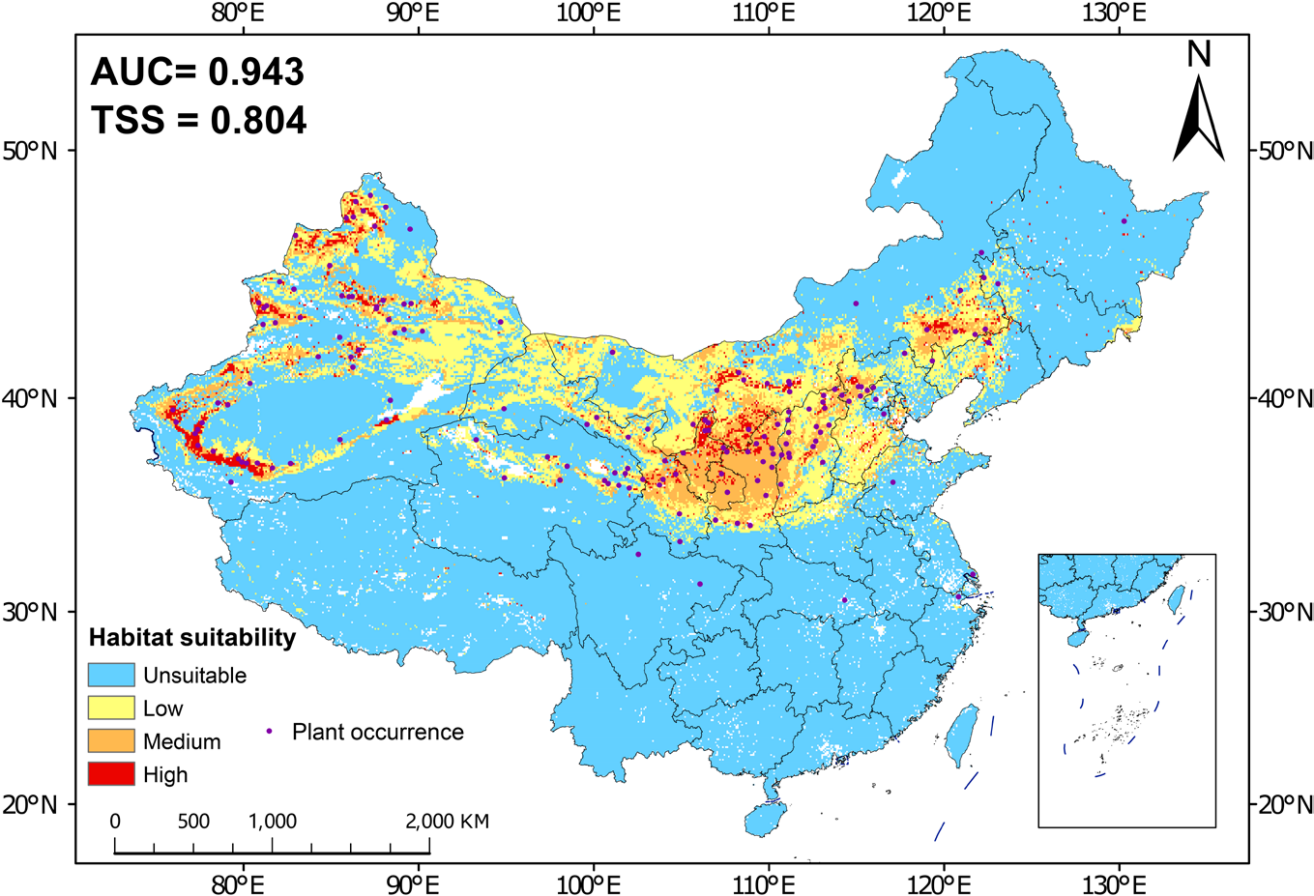
The potential suitable habitats of *Sphaerophysa salsula* species in China under the current climate.

With an average AUC of 0.990 and a TSS of 0.804, the distribution model under the current climate for *S. salsula* demonstrated high accuracy. The habitat suitability model under current climatic conditions showed that nearly all recorded occurrence points of *S. salsula* were located within suitable habitats (Figure 4). The low, moderate, and high suitability zones accounted for 14.6%, 6.4%, and 1.7% of China's total land area, respectively. The moderate‐to‐high suitability areas were primarily distributed in central and western Xinjiang, central and southeastern Inner Mongolia, Ningxia, eastern Gansu, central and northern Shaanxi, western and central Shanxi, central Qinghai, as well as northern and southern Hebei. Low suitability areas occurred predominantly in peripheral regions adjacent to the moderate‐high suitability zones, mainly covering Hebei, eastern Xinjiang, northern Gansu, and central‐western Inner Mongolia.

### 3.5 Impact of Future Climate Scenarios on the Potential Distribution of *S. salsula*

**FIGURE 5:**
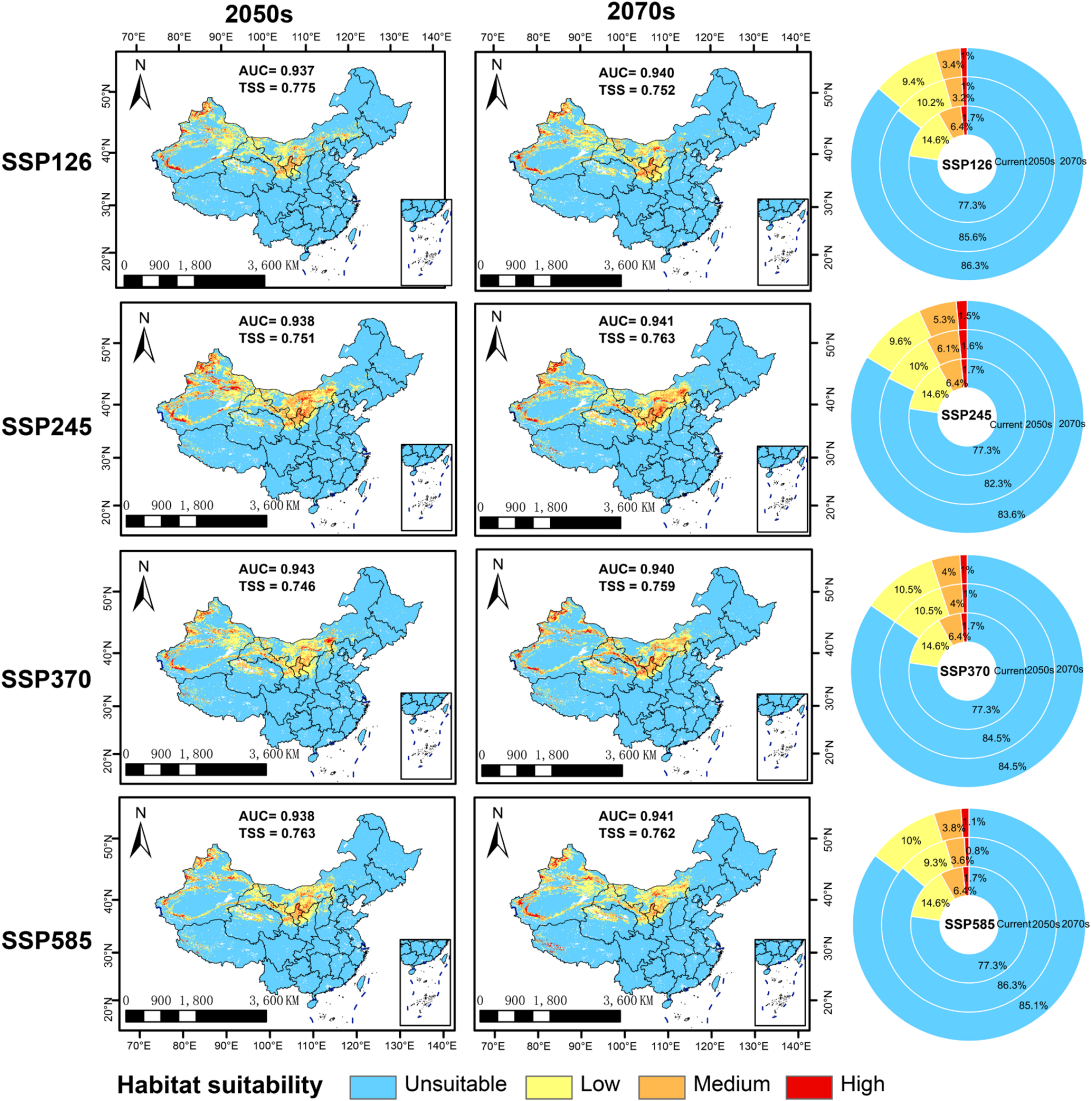
The habitat suitability of *Sphaerophysa salsula* in China under 4 Shared Socioeconomic Pathways in the years 2040s and 2060s.

The MaxEnt model showed consistently high accuracy across all future projections (AUC: 0.937–0.943; TSS: 0.746–0.775) (Figure 5). Under all four future climate scenarios, the suitable habitat of *S. salsula* exhibited consistent declining trends. The most significant reductions occurred from the present to the 2050s (5%–6%), followed by more modest declines from the 2050s to the 2070s (0%–1.2%). Specifically, habitat loss was minimized under SSP245 (6.3% total reduction by the 2070s) and maximized under SSP126 (9% reduction by the 2070s).

**FIGURE 6:**
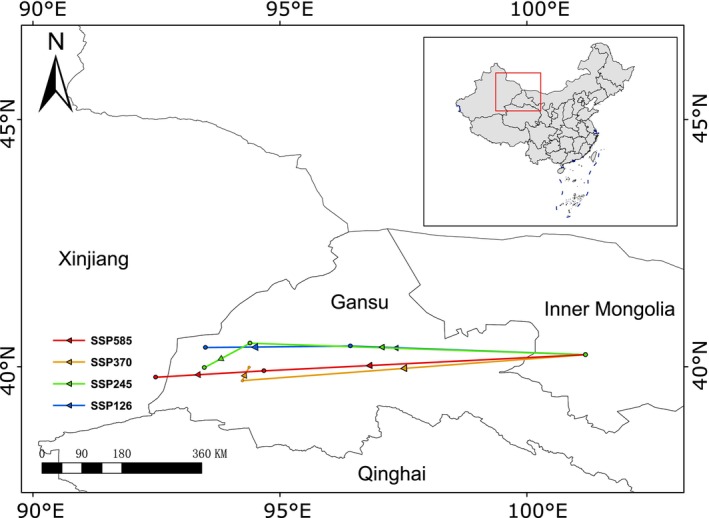
Centroids shift of *Sphaerophysa salsula* on future climate scenarios in the future.

Spatially, the suitable range of *S. salsula* demonstrated a westward shift under all scenarios (Figure 6). By the 2070s, the centroid of habitat shifted from western Inner Mongolia to eastern Xinjiang under SSP585, while the other three scenarios (SSP126, SSP245, and SSP370) showed a westward displacement toward western Gansu.

### 3.6 The Risk of Livestock Poisoning to *S. salsula* in the Current Climate and Future Climate Scenarios

**FIGURE 7:**
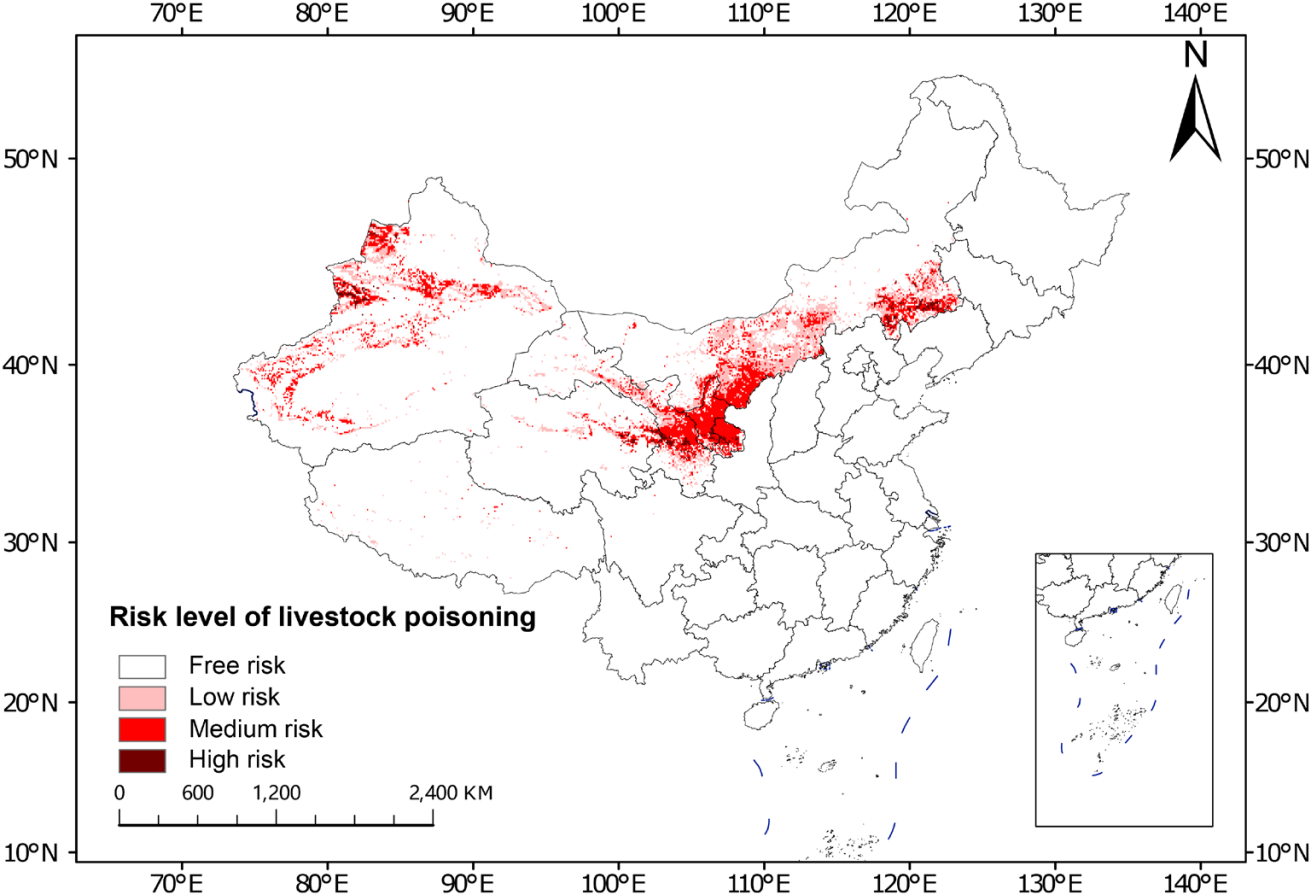
The risk of livestock poisoning to *Sphaerophysa salsula* species in China under the current climate.

Based on grazing intensity and habitat suitability, the current areas with medium/high risk of livestock poisoning due to *S. salsula* were primarily concentrated in Gansu, Ningxia, western Xinjiang, southern Inner Mongolia, and eastern Qinghai (Figure 7). Low poisoning risk distribution primarily surrounded by or was adjacent to the medium/high risk zones, forming a transitional buffer.

**FIGURE 8:**
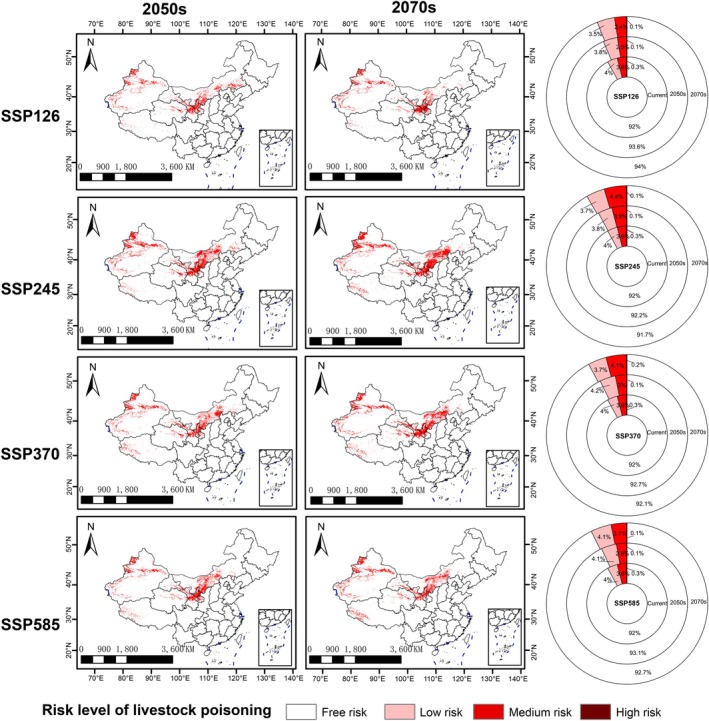
The risk of livestock poisoning to *Sphaerophysa salsula* species in China under 4 Shared Socioeconomic Pathways in the years 2040s and 2060s.

Under future climate scenarios, the high‐risk areas showed a decline, decreasing from 0.3% to 0.1% (Figure 8). Although the total area at risk of poisoning (the combined area of all Low, Medium, and High‐risk zones) exhibited minor fluctuations, the overall change was insignificant. Under the SSP245 scenario, by the 2070s, the risk area increased by 0.3%. In the other three climate scenarios, by the 2070s, the risk area decreased by 0.1% to 2%. The medium‐ and high‐risk zones shifted westward, with a reduction in risk areas in eastern Inner Mongolia and an increase in risk areas in southwestern Tibet.

## 4 Discussion

### 4.1 First Confirmation of Endophyte‐Mediated Swainsonine Toxicity in *S. salsula*

The potential livestock poisoning by *S. salsula* has not been explained (Gao et al. 2012). This study provided the first conclusive evidence that *S. salsula* should be classified as a highly toxic locoweed and that its toxicity originates from the swainsonine‐producing endophyte *A. oxytropis*. Swainsonine, a water‐soluble tri‐hydroxyindolizidine alkaloid, was first identified from *Swainsona canescens* in Australia (Colegate et al. 1979), with subsequent identification in *Astragalus* and *Oxytropis* species (locoweed) across the Americas and Asia (Molyneux and James 1982).

Swainsonine‐containing plants can be divided into two primary chemotypes based on their swainsonine content: chemotype 1 (> 0.1%) and chemotype 2 (< 0.01%), with an intermediate type generally recognized between 0.01% and 0.1% (Cook et al. 2011). Chemotype 1 plants typically exhibit higher endophyte load and greater toxicity compared to chemotype 2 plants (Cook et al. 2011). The main locoweeds in China are chemotype type 1 or intermediate type (Guo et al. 2022), the swainsonine content of which ranged between 0.01% and 1.19% (Table 3). This study found that the swainsonine content of *S. salsula* in Qinghai was 0.373% (mean). This places it within chemotype 1 and at the upper end of the range reported for other locoweeds (0.01%–1.19%). This study also found that *S. salsula* contained endophyte *A. oxytropis*, which could produce swainsonine in vitro. *A. oxytropis* is a known producer of swainsonine—the primary toxin present in legumes (Braun et al. 2003). In addition, the swainsonine content of poisonous *S. salsula* plants was related to the endophyte amounts and the swainsonine concentrations of the corresponding endophyte, similar to locoweed (Grum et al. 2012). *Sphaerophysa* became the fourth legume genus containing swainsonine besides *Astragalus*, *Oxytropis*, and *Swainsona* (Braun et al. 2003; Colegate et al. 1979; Ralphs et al. 2002). Notably, while *Sphaerophysa* is phylogenetically closer to *Swainsona* (which hosts endophyte *A. swainsoniae*) (Neyaz et al. 2025), it harbors the same endophyte species (*A. oxytropis*) as the more distantly related Chinese *Oxytropis* and *Astragalus*. This finding deviates from expectations based on host phylogeny but aligns closely with the pattern of endophyte monodominance observed in Chinese locoweeds. Despite the taxonomic diversity of locoweed hosts in China, *A. oxytropis* is currently the only confirmed *Alternaria* sect. *Undifilum* species (Guo et al. 2022; Yu et al. 2010). In contrast, higher species diversity within *Alternaria* sect. *Undifilum* has been documented on *Astragalus* in America, though notably, *A. oxytropis* similarly appears to be the sole species associated with *Oxytropis* there (Pryor et al. 2009). The dominance of *Oxytropis* among Chinese locoweeds may have provided the ecological basis for the widespread establishment and competitive success of *A. oxytropis*. Through long‐term, specialized co‐evolution with these dominant hosts, *A. oxytropis* likely gained a strong competitive advantage, reinforcing its dominance within the local endophyte community (Fukami 2015). Thus, the endophyte in *S. salsula* reflects not strict host phylogeny, but rather the composition of the regional endophyte community and the ecological context of its coexistence with dominant Chinese locoweeds.

**TABLE 3:** The content of swainsonine in the common locoweed in China.

Species	Swainsonine (% Mean)	Chemotype	References
*Oxytropis glacialis*	1.1900	Chemotype 1	Guo et al. (2022)
*O. deflexa*	0.3267	Chemotype 1	Guo et al. (2022)
*O. sericopetala*	0.2140	Chemotype 1	Guo et al. (2022)
*O. falcata*	0.1643	Chemotype 1	Guo et al. (2022)
*O. glabra*	0.0668	Intermediate	Guo et al. 2022
*O. ochrocephala*	0.0479	Intermediate	Guo et al. (2022)
*O. kansuensis*	0.0145	Intermediate	Guo et al. (2022)
*Astragalus strictus*	0.052	Intermediate	Yu et al. (2010)
*A. variabilis*	0.034	Intermediate	Yu et al. (2010)

*Note:* Plants that contain swainsonine > 0.1%: chemotype 1; plants that contain swainsonine < 0.01%; chemotype 2: chemotype 1. Between the chemotype 1 and 2 are intermediate.

Notably, Gao et al. (2012) detected neither *Alternaria* sect. *Undifilum* nor swainsonine (or only trace amounts < 0.001%) in *S. salsula* populations from Inner Mongolia. This contrasts sharply with our Qinghai findings, showing strong geographic variation in toxicity within the species. Similar spatial variability in swainsonine production has been seen across natural populations of other locoweeds (Davis et al. 2024). The manifestation of toxicity in a population is likely a complex phenotype governed by multiple interacting determinants, including endophyte colonization density (Grum et al. 2012), endophyte genotype (Cook et al. 2013), host genotype (Guo et al. 2022), and local environmental conditions (Klypina et al. 2017). The environmental dependency of this symbiotic relationship is of particular importance for biogeographic risk assessment under climate change (Zhang, Li, and Shi 2025), as it suggests that the spatial distribution of toxic *S. salsula* populations may itself be dynamic and subject to shifts in response to future climatic conditions. Although our study confirmed the high toxicity of *S. salsula* in Qinghai, the significant geographic variability in toxicity underscores the need for further investigation and comprehensive assessment of the species' overall toxicity profile across its entire range.

This spatiotemporal dynamic is further exemplified by seasonal toxicity variation within populations. *S. salsula* in Xinjiang was reported in transition from summer toxicity to autumn palatability post‐frost, becoming a highly addictive forage, which is regarded as high‐quality forage (Qu et al. 2021; Region 1990). This seasonal shift aligns with locoweed as observed by Achata Böttger et al. (2012), who documented a ~50% reduction in swainsonine content between summer and autumn in locoweeds. Although the content of swainsonine decreased after autumn, *S. salsula* in Xinjiang could reflect swainsonine‐induced addictive grazing behavior—a hallmark of locoism (James et al. 1967). Even with Chemotype 2 plants, chronic exposure may still induce reversible adverse effects such as abortion or weight loss (Cook et al. 2011). Consequently, we strongly advise against utilizing *S. salsula* as winter forage in Xinjiang, despite its reported palatability. *S. salsula* has been used for ecological improvement in the United States and China (Deng et al. 2011; Ditomaso and Kyser 2013). Though *S. salsula* affects soil stabilization, it is essential to be vigilant about the potential toxicity risk it poses to livestock (Ditomaso and Kyser 2013).

### 4.2 Changes in the Suitable Habitat of *S. salsula* Under Current and Future Climate Change

Temperature, precipitation, and soil are critical factors influencing plant growth and distribution (Wang, Cheng, et al. 2024; Zhang et al. 2021). This study demonstrates that these environmental variables collectively shape the distribution of *S. salsula* . Key determinants of *S. salsula* habitat suitability included an optimal temperature annual range of 43.22°C, mean temperature of the driest quarter at −6.29°C, pH in water of 8.61, rootable soil depth exceeding 100 cm, and precipitation during the warmest quarter of 239.73 mm. These climatic parameters indicate *S. salsula* adaptation to temperate continental climates, while the soil preferences reveal its deep‐rooted characteristics and tolerance to drought and salt soil conditions. These ecological traits explain its historical use in land rehabilitation programs (Deng et al. 2011). However, given its documented toxicity to livestock, the practical application of *S. salsula* for ecological restoration requires careful reconsideration.

The area under curve (AUC) of the MaxEnt model (0.937–0.943) demonstrated the model's excellent predictive performance. The model predicts that the current suitable habitats for *S. salsula* are primarily located in Xinjiang, Inner Mongolia, Qinghai, Gansu, Ningxia, Shaanxi, Shanxi, Hebei, Liaoning, and Jilin provinces, consistent with historical records (China 1993). Additionally, newly identified suitable areas in northern Henan and northern Shandong suggest a broader potential distribution than previously recognized. As livestock poisoning incidents show a significant positive correlation with habitat suitability (Huang et al. 2022), regions with high suitability warrant particular attention for toxic plant management.

This study highlights that under SSP126, SSP245, SSP370, and SSP585 scenarios, the potential suitable habitat area for *S. salsula* is projected to significantly decline. Global climate change has induced substantial alterations in vegetation growth patterns, morphological characteristics, and distribution ranges (Rosenzweig et al. 2008). Under four future climate scenarios, *S. salsula* habitats are projected to decline by 6.3%–9%, with the most significant reductions occurring in eastern and central regions such as Liaoning, Hebei, Shanxi, and Shaanxi. With global warming, most species' distributions are expected to shift toward higher latitudes and altitudes (Lenoir et al. 2008). The significant shift of *S. salsula* 's suitable habitat center to higher elevation areas in western regions further confirms this pattern (Figure 6). This distributional shift may result from reduced temperature annual range and increased mean temperature of the driest quarter in central and eastern regions caused by global warming, leading to decreased habitat suitability. The western higher‐altitude areas remain less affected by these changes. Consequently, future management efforts should prioritize controlling *S. salsula* in western provinces where grazing activities are frequent.

### 4.3 Changes in the Risk of Livestock Poisoning by *S. salsula* Under Current and Future Climate Change

At present, most model studies related to poisonous plants focus on the construction of suitable habitat models of toxic plants (Xu et al. 2024). However, the risk assessment study on livestock poisoning caused by poisonous plants remains limited. The risk of livestock poisoning is jointly influenced by habitat suitability of poisonous plants and grazing intensity (Holechek 2002; Huang et al. 2022). The research found that the livestock poisoning incidents in the medium and high suitability habitat of poisonous plants was 66% higher than those in the low suitability habitat (Huang et al. 2022), while livestock death losses to poisonous plants under heavy grazing intensities (4.8%) were higher than those under moderate grazing (2.0%) (Holechek 2002). In the present, based on grazing intensity and habitat suitability of *S. salsula* , the risk of livestock poisoning by *S. salsula* under current and future climate change was evaluated.

To our knowledge, this is the first study integrating grazing intensity with SDM outputs to map poisoning risk for toxic plants in China. However, it is essential to clarify that this assessment is built upon several key assumptions, which define both its current limitations and the future directions for model improvement. Firstly, the current risk prediction assigns equal weight to habitat suitability and grazing intensity. This is a practical approach given the lack of geographically referenced poisoning incident data for *S. salsula* . Although the literature suggests that the strength of their influence on risk may differ (Holechek 2002; Huang et al. 2022), directly converting these percentages into weighted scores without validation could introduce new assumptions. Another central assumption of this prediction is that both toxicity and grazing intensity remain relatively stable in the future. However, existing research has shown that the toxicity of locoweeds is regulated by environmental factors such as temperature and precipitation (Guo et al. 2022; Klypina et al. 2017) and exhibits significant geographical heterogeneity (Davis et al. 2024). This suggests that the actual distribution of toxic populations may not fully coincide with the projected suitable habitats. Furthermore, while the spatial pattern of future grazing is likely to shift in response to climate change (Boone et al. 2018), the model employs static, historical average grazing intensity data, which fails to capture the dynamic and adaptive nature of grazing activities.

Despite these uncertainties, which highlight directions for future research, the following risk patterns emerged from our assessment of current and future conditions. This study showed that currently, the areas with risk of livestock poisoning from *S. salsula* in livestock are mainly concentrated in southern Gansu, Ningxia, southern Inner Mongolia, western Xinjiang, and central and western Qinghai. These areas are all traditional pastoral regions (Wang, Peng, et al. 2024), and the presence of *S. salsula* may cause huge economic loss. Moreover, as many pastoral communities in these regions have limited adaptive capacity and high reliance on livestock, the spread of this toxic plant could exacerbate socioeconomic vulnerability and threaten livelihood security (Chen et al. 2015). Under the four future scenarios, although the proportion of areas with poisoning risks fluctuated slightly, the changes were not significant. Although the suitable range of *S. salsula* has declined in future scenarios (Figure 7), this habitat seems to offer limited benefit to the livestock industry. This is because the suitable growth area of *S. salsula* is mainly concentrated in the east. The western pastoral provinces—including Xinjiang, Qinghai, Gansu, Ningxia, and Inner Mongolia—remain within the suitable habitat for *S. salsula* . In addition, the risk of poisoning also showed a trend of spreading to the western regions. Among the current risk areas, the risk of eastern Inner Mongolia has decreased or even disappeared, while western Tibet, which is currently risk‐free, has newly become a risk‐prone area. Livestock are at higher risk of poisoning in areas with fewer plant resources because when there are fewer plants, the probability of livestock consuming toxic plants increases significantly (Li 2009). This dynamic is particularly concerning in ecologically vulnerable regions such as the Tibetan Plateau, where plant productivity is naturally low and ecosystems are sensitive to disturbance (Sun et al. 2020). In such environments, the expansion of *S. salsula* not only introduces a direct toxic threat but may also intensify grazing pressure on remaining palatable vegetation, thereby further increasing the risk of toxic plant ingestion. Therefore, the poisoning risk of *S. salsula* in Tibet deserves special attention from both ecological and pastoral management perspectives.

### 4.4 Conservation Implications: Integrating Biogeographic Risks With Management Solutions

The expansion of potential toxic plants like *S. salsula* under climate change presents complex management challenges, as it requires a delicate balance between leveraging their ecological benefits and mitigating toxicity risks (Davis et al. 2024; Molyneux and James 1982; Smith 1905). Our study confirms that *S. salsula* , historically used as winter forage and for soil stabilization, produces swainsonine, highlighting the urgency of re‐evaluating its utilization (Cook et al. 2021).

In eastern non‐pastoral regions, declines in habitat suitability may lead to reduced abundance or local extinction of *S. salsula* . This may potentially diminish its historical role in soil stabilization and nitrogen enrichment (Deng et al. 2011; Qu et al. 2021; Wang et al. 2021), with implications for local ecosystems in already degraded landscapes. Conversely, its expansion into new western regions such as the Tibetan Plateau introduces a duality of risk and potential influence: while posing a clear toxic threat to livestock, *S. salsula* may also act as an ecosystem modifier, altering soil processes and plant communities. In such regions, particularly the ecologically fragile Tibetan grasslands, proactive monitoring systems must be prioritized to prevent large‐scale livestock poisoning (Sun et al. 2020), even as its potential role in local ecosystem processes is considered.

Therefore, management responses must be spatially differentiated: immediate interventions, including targeted eradication and rotational grazing, are essential in high‐risk zones for poisoning, while suitable areas with low or negligible poisoning risk could potentially be utilized for ecological restoration if endophyte‐free varieties become available (Cook et al. 2016). This case study provides a replicable model for managing similar overlooked toxic species, highlighting the urgent need for toxicity screening, eradication, controlled utilization, and early warning systems (such as drone surveillance and herder‐community‐based monitoring), especially in regions undergoing rapid climate‐induced vegetation changes.

### 4.5 Potential Limitations

Our study has several key limitations. First, the assessment of toxicity in *S. salsula* was based on plant samples from a single population in Qinghai. While the toxic mechanism was successfully confirmed at this site, the known heterogeneity in locoweed toxicity (Davis et al. 2024) necessitates broader surveys across this species' range to determine the overall prevalence of toxic populations. Second, although the changes in the suitable habitat of *S. salsula* were predicted through the MaxEnt model, it only explored factors related to climate and soil due to data limitations, without considering biological factors such as species interactions and human activities. Third, due to data limitations, our poisoning risk evaluation, based on only grazing intensity and habitat suitability, contains inherent simplifications. The model treats both grazing intensity and the plant's toxicity as static variables, overlooking their potential dynamism in response to future climate change (Boone et al. 2018; Guo et al. 2022; Klypina et al. 2017) because of these data gaps. More fundamentally, the risk evaluation assigns equal weight to habitat suitability and grazing intensity. The literature suggests these factors may contribute unequally to poisoning outcomes (Holechek 2002; Huang et al. 2022). The absence of geographically historical poisoning data and toxicity data for *S. salsula* currently precludes a statistically validated, quantitative weighting of these variables. Future versions of this model should aim to integrate dynamic projections of both grazing and toxicity, and to calibrate risk factor weights with empirical poisoning data.

Despite these limitations, the MaxEnt model has been utilized to reliably predict the potentially suitable area and livestock poisoning risk of *S. salsula* in China. Future research will address these limitations to refine the distribution pattern, providing more accurate guidance for the control and utilization of *S. salsula* .

## 5 Conclusions

This study clarified for the first time that field populations of *Sphaerophysa salsula* can function as a high‐toxicity (chemotype 1) locoweed through endophyte‐mediated swainsonine production, and predicted the distribution pattern and livestock poisoning risk by *S. salsula* under climate change using the MaxEnt model. The results indicated that: (1) The toxicity of *S. salsula* is confirmed to originate from swainsonine (0.114%–0.745%) produced by its endophyte *Alternaria oxytropis*, classifying it as highly toxic to livestock; (2) Habitat suitability of *S. salsula* is predominantly influenced by temperature annual range, mean temperature of the driest quarter, soil pH, rootable soil depth, and warmest quarter precipitation; (3) Current suitable habitats of *S. salsula* cover 8.1% of China's territory (predominantly north provinces), and future climate change will lead to habitat contraction, with projected range reductions of 6.3%–9% by the 2070s and a westward shift of habitat center; (4) Currently, the areas with risk of livestock poisoning from *S. salsula* are mainly concentrated in southern Gansu, Ningxia, southern Inner Mongolia, western Xinjiang, and central and western Qinghai, and under future scenarios, the toxicity risk in eastern Inner Mongolia will decrease, while that in western Tibet will increase. These findings highlight the urgent need to re‐evaluate traditional uses of *S. salsula* and similar overlooked toxic plants, particularly as climate change alters their distributions. In addition, the study combines toxicity verification, species distribution modeling, and risk mapping that can be replicated to guide targeted management strategies for mitigating livestock poisoning caused by potential toxic plants. Future research should expand this approach to other suspected toxic species to prevent escalating economic and ecological impacts under global change.

## Author Contributions

**Yue‐Yang Zhang:** conceptualization (lead), data curation (equal), formal analysis (equal), investigation (lead), methodology (equal), resources (equal), software (lead), supervision (equal), visualization (equal), writing – original draft (lead), writing – review and editing (equal). **Hua‐Qi Liu:** methodology (supporting), resources (equal), software (supporting), validation (supporting), writing – review and editing (supporting). **Tong‐Tong Wang:** methodology (equal), resources (equal), software (equal), validation (equal), writing – review and editing (equal). **Ya‐Na Wang:** validation (equal), writing – review and editing (equal). **Yan‐Zhong Li:** conceptualization (equal), funding acquisition (equal), investigation (equal), supervision (equal), validation (equal), writing – review and editing (equal).

## Funding

This research was funded by “National Natural Science Foundation of China (32061123004)”; “National Key R & D Program of China (2022YFD1401103)”; “National Forestry and Grassland Administration (20220104)”; “The Earmarked Fund for CARS (CARS‐34)”.

## Conflicts of Interest

The authors declare no conflicts of interest.

## Supporting information


**Figure S1:** The importance of 31 variables based on the random forest model.
**Figure S2:** Response curve of five key environmental variables.
**Table S1:** Information of newly generated plant sequence and reference sequence in paper.
**Table S2:** Information of newly generated *Alternaria oxytropis* sequence and reference sequence in paper.

## Data Availability

Genetic sequences are deposited in GenBank (www.ncbi.nlm.nih.gov/genbank), and accession numbers were available in the Tables S1 and S2. Occurrence data are available via GBIF (www.gbif.org/occurrence/search?taxon_key=5353525). Environmental layers are from WorldClim (www.worldclim.org/) and Harmonized World Soil Database (https://gaez.fao.org/pages/hwsd). Grazing intensity data are available online at the National Science & Technology Infrastructure (www.nesdc.org.cn).
