# Supplementary material for: Assessing the Biogeographic Risks of Potentially Toxic Plants—A Case Study for a Novel Locoweed Sphaerophysa salsula in China
Source: Ecol Evol. 2026 Feb 11;16(2):e73074. doi: 10.1002/ece3.73074 (PMC12893787; doi:10.1002/ece3.73074)
Supplement: Supplementary file 1 — Figure S1: The importance of 31 variables based on the random forest model. Figure S2: Response curve of five key environmental variables. Table S1: Information of newly generated plant sequence and reference sequence in paper. Table S2: Information of newly generated Alternaria oxytropis sequence and reference sequence in paper. [file ECE3-16-e73074-s001.docx]

**Appendix**

**Assessing the biogeographic risks of** **potentially toxic plants - A case study for a novel locoweed *Sphaerophysa salsula* in China**

*Yue-Yang Zhang ^a^, Hua-Qi Liu* *^a^, Tong-Tong Wang ^a^, Ya-Na Wang ^a^, Yan-Zhong Li ^a,*^*

*^a^ State Key Laboratory of Herbage Improvement and Grassland Agro-Ecosystems, College of Pastoral Agriculture Science and Technology, Center for Grassland Microbiome, Lanzhou University; Engineering Research Center of Grassland Industry, Ministry of Education; Lanzhou 730020, China*

***** Correspondence: Yan-Zhong Li, [liyzh@lzu.edu.cn](mailto:liyzh@lzu.edu.cn)

**Contents**

1. Table S1 Information of newly generated plant sequence and reference sequence in paper

2. Table S2 Information of newly generated *Alternaria oxytropis* sequence and reference sequence in paper

3. Fig. S1 The importance of 31 variables based on the random forest model

4. Fig. S2 Response curve of five key environmental variables

**Table S1** Information of newly generated plant sequence and reference sequence in paper

| GenBank accession numbers | Specimen_spicies | Specimen_voucher |
| --- | --- | --- |
| PV253731* | *Sphaerophysa salsula** | MHLZU6816* |
| OQ804673.1 | *Sphaerophysa kotschyana* | SK-1 |
| LC164587.1 | *Sphaerophysa kotschyana* | GAZI:6585 |
| MH293154.1 | *Oxytropis melanocalyx* | 39666 |
| KJ143722.1 | *Oxytropis falcata* | OL |
| MN871808.1 | *Oxytropis falcata* | MHLZU7478 |
| MT923649.1 | *Sphaerophysa salsula* | TLF-060 |
| MN871862.1 | *Sphaerophysa salsula* | MHLZU6165 |
| MN496359.1 | *Sphaerophysa salsula* | Chang 2010074 (WUK) |
| GQ265958.1 | *Oxytropis glabra* | AZ.4 |
| KJ143719.1 | *Oxytropis glabra* | OG |
| KX955039.1 | *Astragalus tibetanus* | MSB:675 |
| OQ106946.1 | *Astragalus tibetanus* | P291-I1988 |
| KT201387.1 | *Astragalus scaberrimus* | C4 |
| KT201386.1 | *Astragalus scaberrimus* | C3 |
| KT201385.1 | *Astragalus scaberrimus* | C2 |
| MT923550.1 | *Astragalus scabrisetus* | Ge130320 |
| HQ241867.1 | *Astragalus pseudoglaucus* | gla2 |
| MN871856.1 | *Oxytropis kansuensis* | MHLZU7014 |
| MW432237.1 | *Oxytropis kansuensis* | WYJ202008135 |
| MN871827.1 | *Oxytropis ochrocephala* | MHLZU7460 |
| MW432241.1 | *Oxytropis ochrocephala* | WYJ202008394 |
| PP906964.1 | *Sophora alopecuroides* | KS23-3602 |
| MT923616.1 | *Medicago falcata* | Ge130569 |
| KJ143720.1 | *Astragalus variabilis* | AV |
| MN871826.1 | *Astragalus variabilis* | MHLZU7137 |
| GQ246032.1 | *Swainsona canescens* | N G Marchant 20854 (M) |
| GQ246030.1 | *Swainsona flavicarinata* | JR Maconochie 2411 (M) |

***** newly generated plant sequence in paper

**Table S2** Information of newly generated *Alternaria oxytropis* sequence and reference sequence in paper

| ITS | Species | Isolate |
| --- | --- | --- |
| PV242228* | *Alternaria oxytropis** | LYZ0995* |
| PV242229* | *Alternaria oxytropis** | LYZ0996* |
| PV242230* | *Alternaria oxytropis** | LYZ0997* |
| PV242231* | *Alternaria oxytropis** | LYZ0998* |
| PV242232* | *Alternaria oxytropis** | LYZ0999* |
| PV242233* | *Alternaria oxytropis** | LYZ1000* |
| PV242234* | *Alternaria oxytropis** | LYZ1001* |
| PV242235* | *Alternaria oxytropis** | LYZ1002* |
| PV242236* | *Alternaria oxytropis** | LYZ1003* |
| MH861639.1 | *Alternaria abundans* | CBS 534.83 |
| JQ693646.1 | *Alternaria peglionii* | CBS 103.26 |
| JQ693648.1 | *Alternaria alternarina* | EGS 10-193 |
| JQ693663.1 | *Alternaria brassicae* | EGS 38-032 |
| KC584223.1 | *Alternaria vaccariae* | CBS 116533 |
| KC584208.1 | *Alternaria nobilis* | CBS 116490 |
| KP124431.1 | *Alternaria gossypina* | CBS 107.36 |
| AF347031.1 | *Alternaria alternata* | EGS 34-016 |
| KJ718127.1 | *Alternaria calendulae* | CBS 224.76 |
| KJ718143.1 | *Alternaria cirsinoxia* | CBS 113261 |
| AF229475.1 | *Alternaria solani* | ATCC 58177 |
| MH862445.1 | *Alternaria aragakii* | CBS 594.93 |
| MH862449.1 | *Alternaria tropica* | CBS 631.93 |
| KJ718230.1 | *Alternaria sennae* | CBS 477.81 |
| KJ718098.1 | *Alternaria agerati* | CBS 117221 |
| NR_136069.1 | *Alternaria acalyphicola* | CBS 541.94 |
| NR_136070.1 | *Alternaria agripestis* | CBS 577.94 |
| JX827264.1 | *Alternaria fulvum* | DG-2012b |
| FJ357317.1 | *Alternaria bornmuelleri* | DAOM 231361 |
| JX674068.1 | *Alternaria sp.* | ScBT1 |
| JX241634.1 | *Alternaria cinereum* | DG-2012a |
| KJ143731.1 | *Alternaria oxytropis* | OKTZ |
| KP117289.1 | *Alternaria oxytropis* | AS1L4-5 |
| AF229481.1 | *Pleospora tarda* | ATCC 42170 |
| MH861738.1 | *Paradendryphiella salina* | CBS 302.84 |

***** newly generated endophyte sequence in paper


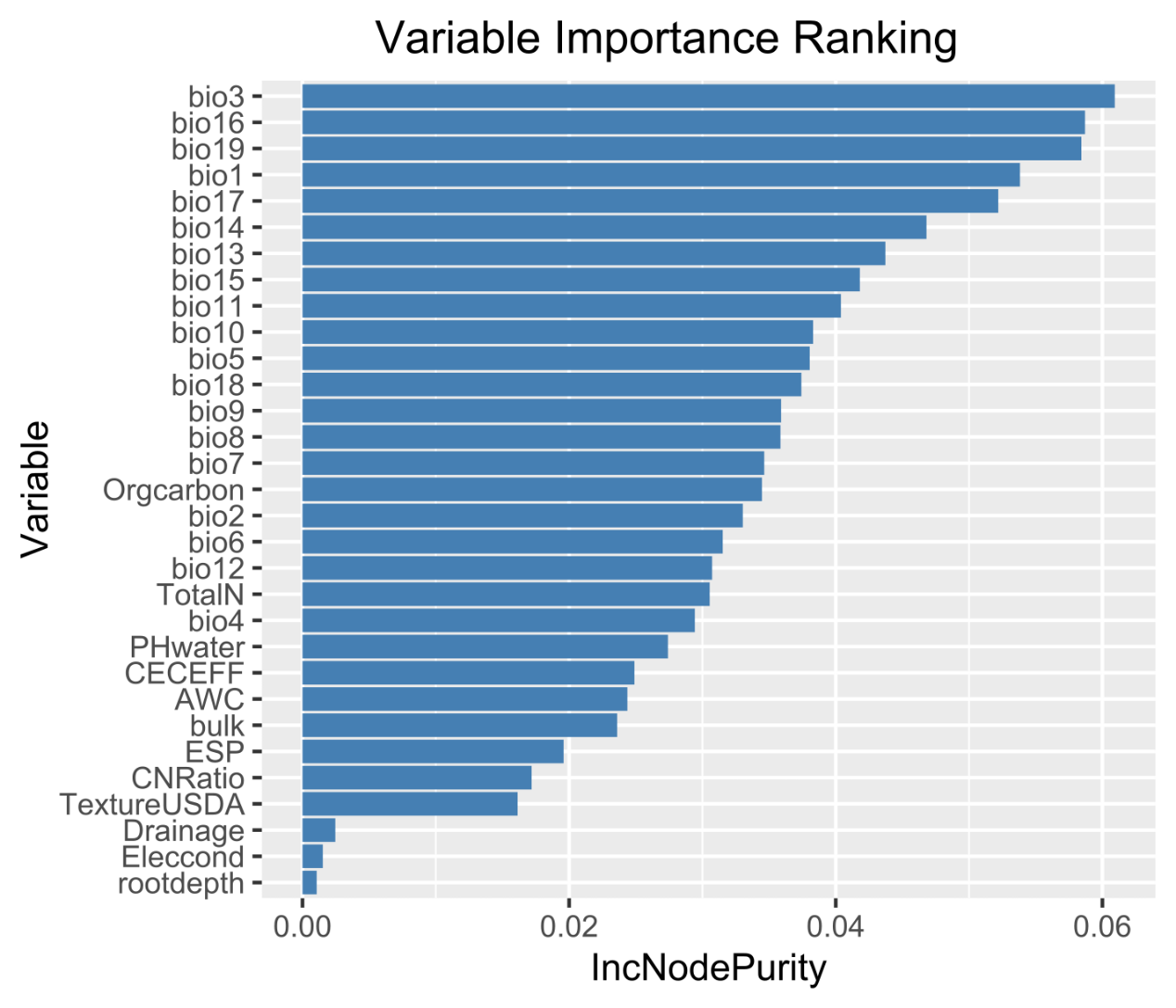


**Fig. S1** The importance of 31 variables based on the random forest model


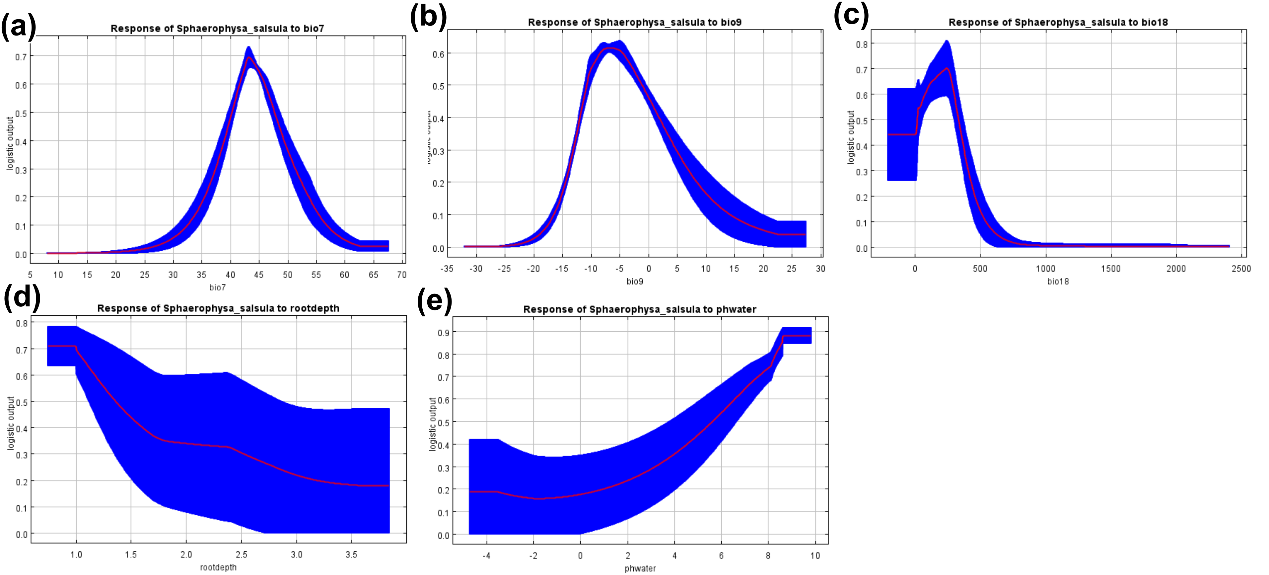


**Fig. S2** Response curve of five key environmental variables
